# Environmental and Occupational Health Exposures and Outcomes of Informal Street Food Vendors in South Africa: A Quasi-Systematic Review

**DOI:** 10.3390/ijerph19031348

**Published:** 2022-01-25

**Authors:** Maasago Mercy Sepadi, Vusumuzi Nkosi

**Affiliations:** 1 Department of Environmental Health, Faculty of Health Sciences, Doornfontein Campus, University of Johannesburg, Johannesburg 2094, South Africa; vusi.nkosi@mrc.ac.za; 2 Environment and Health Research Unit, South African Medical Research Council, Johannesburg 2094, South Africa; 3 Faculty of Health Sciences, School of Health Systems and Public Health, University of Pretoria, Pretoria 001, South Africa

**Keywords:** South Africa, informal, street vendors, environmental health, occupational health

## Abstract

**Introduction:** Informal street vending is a form of informal employment, and occupational conditions for people within this group have been proven to be detrimental to their health. Two independent reviewers carried out a systematic evaluation of the existing literature in South Africa on environmental and occupational exposures, as well as the health effects faced by informal street food vendors. **Methods:** 354 published publications were reviewed and 9 were included, following the Preferred Reporting Items for Systematic Reviews and Meta-Analyses (PRISMA) guidelines. **Results:** The evidence showed that informal street vendors are exposed to workplace risks that affect their health and wellbeing. Non-enclosed vendor stalls and frequent use of open fires were among the risk concerns. Vendors are vulnerable to gastrointestinal diseases such as salmonellosis and respiratory infections such as influenza and COVID-19 due to inadequate access to water, waste disposal facilities, and basic hygiene awareness and practices. Exposure to air pollutants increased the risk of respiratory and urinary illnesses and an impact on the reproductive health of female street vendors. **Conclusions:** This investigation demonstrated the difficulties in complying with the requirements of the Regulations Governing General Hygiene Requirements for Food Premises, the Transport of Food, and Related Matters (no. R638 of 22 June 2018) and the Occupational Health and Safety (OHS) Act (no. 85 of 1993). Within South African borders, there is a scarcity of research on occupational exposures and health effects in this occupation. As a result, eliminating or preventing these occupational exposures should be at the top of government and stakeholder agendas. The majority of the research was carried out in KwaZulu Natal and used a quantitative, cross-sectional technique. Other designs, including cohorts, time series, and randomized intervention trials, were underutilized.

## 1. Introduction

When it comes to finding decent work, there is still a global challenge. According to the current literature, people are engaged in informal labor due to a lack of opportunities, unemployment, poverty, and other factors [1,2]. This emphasizes the importance of addressing the issue of informality in the employment sector, which is estimated to be around two billion people worldwide, including people aged 15 and over [1]. Informal employment is most prevalent in Africa, Arab States, Asia, and the Pacific [1]. Informal sector activities include the retailing of goods or the provision of services solely for the purpose of employment and income generation for the individual involved [1].

Street vending is an activity that provides easy access to a variety of services or goods in public spaces, such as clothing, electronics, traditional medicines, fresh vegetables, and prepared foods [2]. Street vendors play an important role in many countries’ urban economies, and they account for 15% of total urban employment in South Africa (SA) [3]. This activity is prevalent in SA’s big cities such as Johannesburg, Tshwane, Cape Town, and Durban, particularly in the inner cities where industries, businesses, and educational institutions are located. The most important activities in the informal food sector are food production, catering, and sales of fresh or prepared products [4], whereas catering (preparing and cooking food) is a common practice among street vendors and has been noted to generate the most income [5]. However, Department of Health legislations have the greatest impact on informal vendors involved in food preparation [6].

Section 6A of the SA Businesses Act 71 of 1991 [6,7] delegated authority to local governments or municipal entities in SA to manage street trading. Thus, food street vendors must obtain a trading license under Schedule 1 (Items 1 and 3) for the sale, supply, and hawking of meals or perishable foodstuffs. The other health legislations in SA that could affect or may guide informal food vendors include the Foodstuffs, Cosmetics and Disinfectants (FCD) Act (Act no. 54 of 1972): Regulations Governing General Hygiene Requirements for Food Premises, Transport of Food and Related Matters (No R638 of 22 June 2018) [8] and workplace health and safety which could be guided by the Occupational Health and Safety (OHS) Act (Act No. 83 of 1993) [9]. Those informal vendors who work inside formal buildings will also be guided by the National Building Regulations and Building Standards Act 103 of 1977 [10]. 

Informal street vendors face several occupational challenges, including a lack of infrastructure, long working hours, ambient air pollution, and a lack of health and hygiene knowledge for the activities in which they engage. The SA Basic Conditions of Employment Act (Act No. 75.1997) stated a maximum of 45 hours of work time per week (which is nine hours a day for those working five days a week, and eight hours for those working more than five days a week) [11]. However, street vendors in SA appear to work more than 8 hours per day, over seven days a week and 12 months a year [12]. Longer working hours are known to have long-term negative effects on human health and have been linked to increased mental health issues, decreased quality and quantity of sleep, lower back injury, higher blood pressure, and women experiencing an increased risk of spontaneous abortion, lower birth weight, or gestational age [13,14,15].

### The Significance of This Systematic Review

The income of informal workers is determined by their availability at work daily [16]. These workers are frequently subjected to deplorable and hazardous living and working conditions [16]. This review addresses one of the critical discussions in environmental and occupational health research and public health policy-making platforms; it identifies risks, health outcomes, and solutions to the current problems faced by informal workers, including informal street vendors [17,18]. Furthermore, there is a need for environmental and occupational health professionals to intervene in enforcing health legislation and compacting the ill health for these workers. 

“What health risk factors exist in the informal street food vending occupation?” is the primary review question. The secondary review question is “What are the associated health outcomes from the identified risk factors in the informal street trading occupation?”.

The evidence from this review could then be used to more confidently motivate environmental and occupational health professionals and policymakers to analyze the street vending occupation, thus strengthening the current evidence on the occupational risks faced by street vendors and the burden of disease associated with exposure to this occupation in SA.

## 2. Methods

### 2.1. Inclusion and Exclusion Criteria

The methodology discussion in this paper is of pooled studies which are focused on the occupational exposures and health outcomes of informal vendors. The review of the literature was carried out by two independent individuals, and the search was conducted between June 2021 and October 2021.

The inclusion criterion was limited to primary studies conducted in SA. We included all studies that provided environmental and occupational exposures or risk factors, as well as health outcomes of informal street vendors. The review was limited to studies published in English with full text between 2012 and 2021 (within 10 years). Any studies that did not investigate the exposures and health outcomes faced by informal street vendors in SA, as well as those published more than ten years ago, were excluded.

### 2.2. Study Selection Strategy 

**Figure 1:**
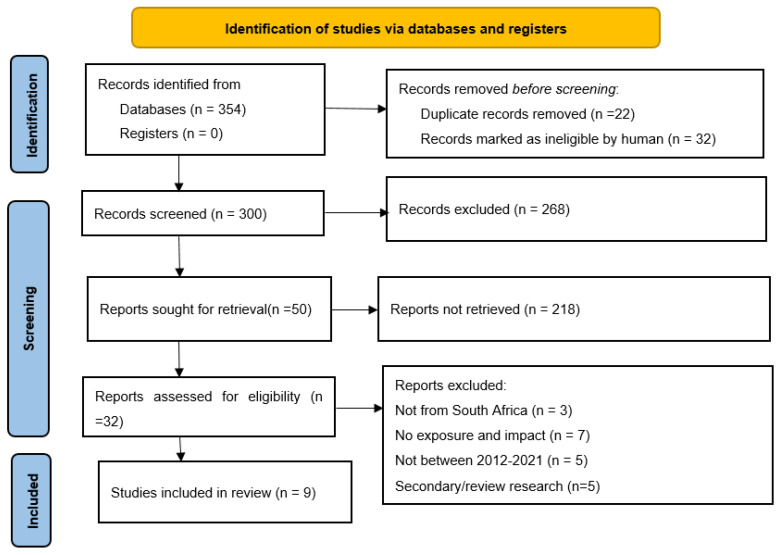
Preferred reporting items for systematic reviews and meta-analyses (PRISMA) flow diagram.

Search results were screened following preferred reporting items for systematic reviews and meta-analysis (PRISMA) [19]. Table 1 and Figure 1 show the four stages of the selection process. Google Scholar, Science Direct, and PubMed were among the electronic databases and journals searched. The first stage involved the use of keywords developed based on the review objectives, problem, population, and outcome (Table 1), with term groups used for separate searches and in various combinations. A total of 354 articles were discovered in the first stage (keyword screening), 322 in the second stage (title and abstract), but only 32 were found to be relevant. The third stage was the screening of full text, and only nine met the criteria for this paper’s results review (Figure 1).

### 2.3. Reviewing Descriptive and Narrative Analysis

The authors set out to find studies that had identified and measured workplace risk factors or exposures and simultaneously measured health outcomes. However, only a few studies were identified related to the review, and only two out of the nine measured both exposure and outcome. Therefore, the authors opted to use descriptive analysis to highlight the summary of the dataset across all studies. A full meta-analysis of the selected studies’ results was also not possible due to the small number of studies found in SA, and differences in the studies’ data collection and results discussion methods. 

## 3. Results 

This section reveals the reviewed results from the selected studies, as well as their interpretation and conclusions. Four of the nine studies were master’s theses that had not been peer-reviewed. Furthermore, data collection was hampered by a few of studies that included vendors who did not sell food.

### 3.1. Study Design 

The results reflect that 89% (*n* = 8) of the studies were conducted following a quantitative, cross-sectional research approach and only 11% (*n* = 1) studies used a qualitative research approach. In this review, only study 8 and 9 (Table 2) measured both the exposure and health outcomes. None of the studies chosen accounted for workers’ work duration, which is an important factor in determining health risks.

### 3.2. Demographic Information of the Studies

The nine selected studies (Table 2) were published between 2012 and 2021, with seven focusing on general hygiene in accordance with Regulation R638 of 22 June 2018 [20,21,22,23,24,25,26,28], and two focusing on exposure and health outcomes among street vendors [5,27,29]. SA has nine provinces in total, but no relevant studies from the Northern Cape, Eastern Cape, Northwest, Free State, or Mpumalanga were found. The majority of the selected studies (44%; *n* = 4) were conducted in KwaZulu Natal (KZN), followed by the Western Cape (WC) (22%; *n* = 2) and Limpopo province (LP) (22%; *n* = 2), with Gauteng province (GP) having the fewest (11%; *n* = 1).

The health risks faced by street vendors are influenced not only by their working environment, but also by socioeconomic statuses such as gender. According to the International Labour Organization (ILO), out of the 2 billion workers in informal employment, men (63.0%) outnumber women (58.1%) globally [1]. Females made up 666 (65%) of the total sample size of 1028 street vendors from the combined nine studies (Table 2), compared to male (*n* = 362; 35%) street vendors. However, as stated in Table 2, Study 9’s sample was limited to female street vendors only. When compared to ILO gender statistics, the gender results of this review show that there are more females than males. Moreover, due to street trading being one of the sectors entered due to unemployment, the 2nd Quarterly Labour Force Survey conducted in 2021 revealed that the unemployment rate among male workers in SA was lower (32, 4%) than that of females (36, 8%) [3], which may support this review’s results of females getting into informal work. Furthermore, the gender results of the review are supported further by how street trading is observed or to be associated with flexibility for women as opposed to men [13,30].

### 3.3. Environmental and Occupational Hazards Faced by Street Vendors

The environment makes a major contribution to public survival in terms of health, and this includes places of work. There are two types of informal vendors, each with their own set of challenges. Street or outdoor workplaces with no formal building structure, such as tents, boxes, or mobile food trailers, are common for informal vendors. These vendors operate in public spaces (for example, a storefront, sidewalks or pavements, public outdoor markets) near downtown streets, markets, or parks [31]. However, some informal vendors occupy markets in discreet locations in an enclosed permanent brick structure. These vendors may operate in semi-public spaces, such as inside market buildings and public transportation stations [31], such as train stations, taxi ranks, and so on. According to this review’s findings (8 out of 9 studies), most street vendors occupy non-permanent shelters on the roadside. The majority of the studies’ samples (8 out of 9) had food trading vendors, with the majority of them participating in the cooking activities. Then the lowest number of street vendors were those who offer non-food services or goods. Most vendors used fire to cook, with only a few using electrical or gas stoves, according to the studies that included the method of cooking in their results (7 out of 9).

Other difficulties encountered by street vendors, as noted by Studies 1 to 7 (Table 2), included a lack of access to services such as water, ablutions, and waste disposal facilities, as well as the use of communal water points as opposed to those found with water within their stalls. Furthermore the littering caused by street vendors. According to Table 2, all the general hygiene and food vendor-related studies (Studies 1 to 7) revealed that many street vendors are untrained or uninformed about food and personal hygiene and safety, such as hand washing, which is an important key factor for food handlers. Additionally, many vendors were discovered to be without their protective clothing.

### 3.4. Occupational Health Outcomes amongst Street Vendors

Only Studies 8 and 9 (Table 2) of the nine chosen studies reflected both exposure and health outcomes, and these two studies were only concerned with illnesses caused by ambient air pollution. The results of a respiratory health impact review (from Study 9) revealed that street vendors exposed to ambient air pollution had a higher risk of developing chronic bronchitis and had lower lung capacity than non-exposed street vendors (Table 2). According to Study 8 (Table 2), the amount of time spent near wood, as well as the amount of wood used for cooking, had a weak positive association with urinary levels of arsenic (As), chromium (Cr), and copper (Cu) among street vendors.

## 4. Discussion 

The few published studies in SA in recent years have demonstrated the associations between various occupational risk factors and health outcomes among street vendors. This review’s findings on the type of street vending stalls support the current international literature, which highlights various infrastructural challenges faced by informal vendors, such as a lack of proper stall shelter and equipment, limited access to electricity, and a lack of access to water, sanitation, and ablution facilities, as well as improper waste management [14,15]. Furthermore, there is a risk associated with outdoor work environments. Informal vendors working in open public spaces, particularly those in informal structures with limited coverage or stalls or workplaces that are not properly weather-proofed, are more vulnerable to environmental hazards than those working in enclosed vendor stalls [7,20,32].

The review results revealed a higher proportion of cooking vendors. Most cooking and food preparation vendors prefer to use less expensive, more accessible fuel sources, such as biomass fuels [20,33]. In SA, there has been an increase in the use of Mbuwula, or “a brazier” by street vendors, which burns coal, wood, and kerosene. While Charcoal, biomass, and kerosene fuels are classified as high-pollution fuels, and electricity and liquid petroleum gas are classified as low pollution fuels [33]. The use of biomass fuel as a heating source may pose a health risk to this group of workers.

More risk factors identified by this review were the lack of waste bins and street vendors including food vendors’ food preparation and serving activities, which results in littering. Another issue is that food vendors discharge wastewater onto the pavements. These vendors’ actions littering is a violation of the various SA municipalities’ public health and street trading by-laws. These laws stipulate requirements such as keeping your trading area clean and ensuring that no wastewater, smoke, fumes, etc., from trading activities pollute the environment or cause harm to the public. Personal behavior is critical in lowering the risks associated with human health. 

The lack of Personal Protective Equipment, the impossibility of implementing physical distancing due to a lack of space, as well as inadequate access to water and sanitation, all have an impact on the informal vendors’ ability to follow COVID-19 prevention guidelines [7,23,24]. The same effect was observed in Ghana, where street vendors reported a lack of hand sanitizers and water in markets [7,23,24]. Furthermore, contaminated hands increase the risk of respiratory infections such as influenza, colds, and COVID-19 [34,35]. Because most street vendors rely on communal water facilities, they are vulnerable to general hygiene-related illnesses due to a lack of hand-hygiene practices and general stall cleanliness. Contaminated food and water caused diarrheal diseases which have been estimated to result in 1.8 million deaths per year, hence a need for proper food preparation and preparation of food in hygienic places to prevent most foodborne diseases [25]. It is estimated that hand washing with soap and water could reduce diarrheal-disease-associated deaths by up to 50%, and reduce the risk of respiratory infections by 16% [34,35].

Ambient air pollution has been linked to upper and lower respiratory health symptoms and diseases among informal street vendors, as well as an increased risk to reproductive health. Because some women choose to continue working in the street trading environment while pregnant, it has been observed that this has an impact on the health of their unborn children. 

The findings of this review are similar to those of a study conducted in Accra, Ghana, which found that street vendors’ exposure to PM_2.5_ escalates the incidence of respiratory and cardiovascular symptoms [36]. Furthermore exposure to high-pollution fuels was associated with low infant birth weights in female street vendors [33,36,37]; additionally, increased likelihood of coughing, postnasal drip, sneezing, rapid or irregular heart beating, sharp chest pains, fainting spells, headaches, and dizziness were linked to increase PM_2.5_ [36].

## 5. Limitations

There is clear scientific evidence from numerous authors worldwide who have investigated informal vendors and the negative health exposure impacts in their occupational settings [37,38,39,40,41,42,43,44,45,46]; however, several limitations must be considered for the objectives of this review article. There were very few studies in SA to critically review, and the current literature has a limitation in that most studies did not focus on identifying both hazards and resulting effects. The shortfall is that only two studies reviewed in this paper were studies on exposures and health outcomes. The findings of the studies were difficult to meta-analyze due to the heterogeneity in how data were collected from street vendors. Hence, the researchers instead opted for descriptive analysis.

## 6. Conclusions

The studies on environmental and occupational health hazards and outcomes in SA provide some evidence on the health impact faced in street vending activity. During the COVID-19 pandemic, we deepened our understanding of the importance of informal food vendors in food systems, and the reason many street vendors continued to work, putting their health at risk [2,47,48,49]. Informal food vendors are an important part of the food system that must be addressed by all health and safety enforcement agencies. The identified risk factors from this review revealed a lack of compliance with SA health legislation. According to the health outcomes, exposure to air pollution increases the risk of respiratory health problems, urinary infections, and reproductive health problems. Furthermore, a lack of hygiene knowledge or practices such hand washing may increase the risk of respiratory infections such as influenza, COVID-19, and gastrointestinal infections.

An increase in informality in all its forms is a major challenge for long-term development. Informality is detrimental to workers’ rights, including fundamental principles and rights at work, as well as social protection [1]. The current state of government involvement in street vendors includes economic development, street by-law departments that focus on vendor location and operation, and environmental health departments that focus on food safety and environmental nuisances. Various non-governmental organizations have had some success in publishing the working conditions of street vendors in SA. Street vendors reported difficulties due to limited access to municipal services such as water availability and accessibility, as well as waste management. Due to a lack of access to water, proper food preparation equipment, and wash-up facilities; informal street vendors face difficulties complying with various local governments’ regulations, such as street trading by-laws and regulations R638 of 22 June 2018, which focus on public health and ensuring food safety, as well as food premises’ structural compliance. The lack of legislation or policy support and implementation has had some negative impact on the protection of street vendors’ health in their occupation.

In conclusion, this review revealed that occupational exposures and health effects of street vendors are under-researched in SA. This literature review is of significant in SA where informal trading is one of the main pillars of the economy, and the environmental and occupational health hazards may increase the morbidity of ill health among these workers. The government and non-governmental organizations should address environmental and occupational health risks, thereby committing to achieving decent work, good health, and wellbeing for all. Furthermore, current health and safety policies and implementation strategies must be re-evaluated.

## Figures and Tables

**Table 1:** Review keyword literature search.

Hazards/Risks		Outcome	Population	Combinations of Keywords
Keywords—Group 1	Keywords—Group 2	Keywords—Group 3	Keywords—Group 4	
Environmental risks; Occupational risks	Infrastructure; general hygiene	Health outcomes; effects on health	Informal OR street food traders or vendors OR street food vending in South Africa	Merge keywords from Group 1, 2, and 3 with keywords from group 4 until all combinations are exhausted

**Table 2:** Environmental and occupational exposures of informal street vendors and their associated health outcomes.

Author(s) (Year)	Population/Location	Study Design	Sample SIZE	Hazard/Exposure	Health Outcome
**1.** **Rohith (2021) [20]**	Western Cape, South Africa	Quantitative cross-sectional	25 street vendors	Lack of general hygiene; Lack of infrastructure (ablution, waste disposal and water facilities); Not adhering to Personal Protective Equipment	Not collected
**2.** **Nkosi (2020) [21]**	Kwa-Zulu Natal, South Africa	Quantitative cross-sectional	400 street vendors	Lack of general hygiene; Lack of infrastructure (ablution, waste disposal and water facilities); Not adhering to Personal Protective Equipment	Not collected
**3.** **Khuluse (2020) [22]**	Kwa-Zulu Natal, South Africa	Quantitative cross-sectional	54 street vendors	Lack of general hygiene; Lack of infrastructure (ablution, waste disposal and water facilities); Not adhering to Personal Protective Equipment	Not collected
**4.** **Tleane (2020) [23]**	Limpopo, South Africa	Quantitative cross-sectional	20 street vendors	Lack of general hygiene; Lack of infrastructure (ablution, waste disposal and water facilities); Not adhering to Personal Protective Equipment	Not collected
**5.** **Mukwevho (2018) [24]**	Limpopo, South Africa	Quantitative cross-sectional	155 street vendors	Lack of general hygiene; Lack of infrastructure (ablution, waste disposal and water facilities); Not adhering to Personal Protective Equipment	Not collected
**6.** **Mjoka et al. (2017) [25]**	Kwa-Zulu Natal, South Africa	Qualitative	19 street vendors	Lack of general hygiene; Lack of infrastructure (ablution, waste disposal and water facilities); Not adhering to Personal Protective Equipment	Not collected
**7.** **Oladipo-Adekeye, Tabit (2021) [26]**	Gauteng, South Africa	Quantitative cross-sectional	315 street vendors	Lack of general hygiene; Lack of infrastructure (ablution, waste disposal and water facilities); Not adhering to Personal Protective Equipment	Not collected
**8.** **Dalvie et al., (2015) [27]**	Western Cape, South Africa	Quantitative cross-sectional	40 street vendors	Clinical assessment, wood assessment	Ambient pollutant (operating braai-stand/using wood with chromated copper arsenate)
**9.** **Hariparsad, S. 2016 [5]; Hariparsad, S., Naidoo 2019 [28] ^1^**	Kwa-Zulu Natal, South Africa	Cross-sectional	305 female vendors	questionnaires, clinical assessments, and lung function tests	Respiratory (risk of developing chronic bronchitis, decreased lung capacity);
					Reproductive (risk of having a low-birth-weight infant, 3 times more likely to be infertile)

^1^ Study no. 9 has two published articles with the same sample size group.

## Data Availability

All data in this study were provided in the main manuscript.
